# mRNA-Lipid Nanoparticle (LNP) Delivery of Humanized EpCAM-CD3 Bispecific Antibody Significantly Blocks Colorectal Cancer Tumor Growth

**DOI:** 10.3390/cancers15102860

**Published:** 2023-05-22

**Authors:** Vita Golubovskaya, John Sienkiewicz, Jinying Sun, Yanwei Huang, Liang Hu, Hua Zhou, Hizkia Harto, Shirley Xu, Robert Berahovich, Walter Bodmer, Lijun Wu

**Affiliations:** 1 Promab Biotechnologies, 2600 Hilltop Drive, Richmond, CA 94806, USAliang.hu@promab.com (L.H.);; 2 Cancer & Immunogenetics Laboratory, Weatherall Institute of Molecular Medicine, John Radcliffe Hospital, Oxford OX3 9DS, UK; 3 Forevertek Biotechnology, Janshan Road, Changsha Hi-Tech Industrial Development Zone, Changsha 410205, China

**Keywords:** colorectal cancer, EpCAM, CD3, bispecific antibody

## Abstract

**Simple Summary:**

Colorectal cancer is one of the most common cancers worldwide, and novel treatments are urgently needed to improve treatment for cancer patients. In this report, three different designs of humanized EpCAM-CD3 antibodies were engineered and tested against colorectal tumors. The antibodies demonstrated high efficacy and specificity. In addition, the study demonstrates a novel method of delivering bispecific antibodies using mRNA-lipid nanoparticle (LNP) technology. The delivery of EpCAM-CD3 human Fc (hFc) mRNA-LNPs into mice tumors with intravenous T cell injection significantly blocked OVCAR-5 xenograft tumor growth in vivo. The data provide a basis for future clinical studies.

**Abstract:**

The epithelial cell adhesion molecule (EpCAM) is often overexpressed in many types of tumors, including colorectal cancer. We sequenced and humanized an EpCAM mouse antibody and used it to develop bispecific EpCAM-CD3 antibodies. Three different designs were used to generate bispecific antibodies such as EpCAM-CD3 CrossMab knob-in-hole, EpCAM ScFv-CD3 ScFv (BITE), and EpCAM ScFv-CD3 ScFv-human Fc designs. These antibody designs showed strong and specific binding to the EpCAM-positive Lovo cell line and T cells, specifically killed EpCAM-positive Lovo cells and not EpCAM-negative Colo741 cells in the presence of T cells, and increased T cells’ IFN-gamma secretion in a dose-dependent manner. In addition, transfection of HEK-293 cells with EpCAM ScFv-CD3 ScFv human Fc mRNA-LNPs resulted in antibody secretion that killed Lovo cells and did not kill EpCAM-negative Colo741 cells. The antibody increased IFN-gamma secretion against Lovo target cells and did not increase it against Colo741 target cells. EpCAM-CD3 hFc mRNA-LNP transfection of several cancer cell lines (A1847, C30, OVCAR-5) also demonstrated functional bispecific antibody secretion. In addition, intratumoral delivery of the EpCAM-CD3 human Fc mRNA-LNPs into OVCAR-5 tumor xenografts combined with intravenous injection of T cells significantly blocked xenograft tumor growth. Thus, EpCAM-CD3 hFc mRNA-LNP delivery to tumor cells shows strong potential for future clinical studies.

## 1. Introduction

Colorectal cancer is one of the most common cancers worldwide [1,2]. Patients with metastatic colorectal cancer will typically have a poor prognosis and a low survival rate. Recently, different approaches for colorectal cancer therapy have been developed such as monoclonal antibodies, vaccines, checkpoint inhibitors, oncolytic viruses, and CAR-T cell therapy [3,4]. Novel therapeutic approaches are urgently needed to improve treatment for patients with colorectal cancer.

One of the attractive targets for immunotherapy is EpCAM (CD326), a 39–40 kDa human cell surface glycoprotein, which is highly expressed in many cancer cells such as ovarian, breast, colorectal, prostate, lung, pancreatic, and others [5]. EpCAM was discovered over 40 years ago as an epithelial antigen and was later classified as a cell adhesion molecule [6]. EpCAM plays an important role in colorectal cancer biology and is also involved in survival signaling, motility, differentiation, cell proliferation, adhesion, and metastasis [6,7,8]. EpCAM can be expressed not only in epithelial cells but also in the stem cells of different tissues and in embryonic stem cells [9]. Recently, the EpCAM AUA1 antibody was used to detect circulating tumor cells, which can be used for the early detection of many carcinomas [10].

EpCAM shows high potential as a target for developing anticancer therapies and immunotherapies with monoclonal antibodies or bispecific antibodies. Recent studies have targeted EpCAM with monoclonal antibodies [11], bispecific antibodies, immunotherapy [12], siRNA, and CAR-T cells [5,13,14,15,16].

Recently, lipid nanoparticles (LNPs) were used for the delivery of the COVID-19 mRNA vaccine by the biopharmaceutical companies Moderna and Pfizer [17,18,19]. The nanoparticle-based drug delivery demonstrated many advantages, such as high bioavailability, solubility, stability, passage through the blood–brain barrier, and low toxicity and minimal side effects [20].

In this study, mRNA-LNPs were used to deliver EpCAM-CD3 bispecific antibodies to tumor sites, stimulating T cells to kill colorectal tumors. Three different formats of novel bispecific antibodies based on the humanized AUA1 antibody [10] were engineered for this study. All bispecific antibodies demonstrated high efficacy and specificity with the EpCAM-positive Lovo cell line but not with the EpCAM-negative Colo741 cell line. In addition, we designed EpCAM-CD3 hFc mRNA, which was embedded into an LNP complex, and showed that the secreted antibodies had highly specific and effective killing activity when combined with T cells and induced the secretion of IFN-gamma in a dose-dependent manner against the EpCAM-positive target colorectal cell line. We also showed that OVCAR-5, A1847, and C30 cancer cells transfected with EpCAM-CD3 mRNA-LNPs secreted EpCAM-CD3 antibodies with high cytotoxic activity. In addition, EpCAM-CD3 hFc mRNA-LNP delivery to OVCAR-5 xenograft tumors with the intravenous injection of T cells demonstrated a significant decrease in tumor growth in vivo. These data provide a basis for the generation of EpCAM-CD3 antibodies in vivo through the mRNA-LNP delivery platform. The produced EpCAM-CD3 hFc antibody shows high antitumor efficacy and potential for use in clinical studies.

## 2. Materials and Methods

### 2.1. Cell Lines

The HEK-293, A1847, C30, OVCAR-5, HeLa, Hep3B, SKOV-3, and PC3 cell lines were obtained from ATCC. The A1847, C30, HeLa, Hep3B, and OVCAR-5 cells were cultured in Dulbecco’s modified Eagle’s medium (DMEM) medium with 10% FBS and penicillin/streptomycin. The PC3 cell line was cultured in an F-12K medium with 10% FBS and penicillin/streptomycin. The SKOV-3 cell line was cultured in a McCoy’s 5A medium with 10% FBS and penicillin/streptomycin. Lovo and Colo741 cell lines were obtained from Dr. Walter Bodmer (Oxford University, Oxford, UK), whose laboratory authenticated the cell lines using SNPs, Sequenom MassARRAY iPLEX, and HumanOmniExpress-24 BeadChip arrays, and tested for the absence of Mycoplasma [21]. The Lovo cell line was cultured in Ham’s F-12 K Medium with 10% FBS and penicillin/streptomycin. The Colo741 cell line was cultured in an RPMI-1640 medium with 10% FBS and penicillin/streptomycin. The Lovo cell line with knockout of EpCAM (Lovo EpCAM KO) was generated using electroporation of the EpCAM sgRNA kit from Synthego and caspase-9 protein following the manufacturer’s protocol. The HEK-293 suspension cells were cultured in a FreeStyle™ F17 expression medium and supplemented with Gibco™ GlutaMAX™ and Pluronic™ F-68 nonionic surfactant (100×). The cell lines were authenticated by FACS using specific cell surface marker detection antibodies. Human peripheral blood mononuclear cells (PBMCs) were isolated from whole blood from the Stanford Hospital Blood Center according to the IRB-approved protocol (#13942). PBMCs were isolated using density sedimentation over Ficoll-Paque (GE Healthcare, Chicago, IL, USA) [22,23,24,25]. PBMCs were suspended at 1 × 10^6^ cells/mL in an AIM-V medium (ThermoFisher, Waltham, MA, USA) containing 10% FBS and 10 ng/mL IL-2 (ThermoFisher), mixed with CD3/CD28 Dynabeads (ThermoFisher) at a 1:1 ratio, and cultured for 10–12 days in 24-well plates. Fresh medium with 10 ng/mL IL-2 was added every 2–3 days to maintain the PBMC cell number at 1–2 × 10^6^ cells/mL. All cell lines and PBMCs were cultured in a humidified 5% CO_2_ incubator. 

### 2.2. Antibodies

APC antihuman CD326 (EpCAM) antibody was obtained from Biolegend (Cat No.: 324207). PE-conjugated anti-His tag antibody was from Biolegend (Cat. No.: 362603). APC antihuman CD3 antibody was obtained from Biolegend (Cat No.: 317318). Antihuman IgG was from Jackson ImmunoResearch (109-605-190). PE streptavidin was obtained from Biolegend (Cat. No.: 405204) and 7-AAD viability staining solution was obtained from Biolegend (Cat. No.: 420404).

### 2.3. Design and Cloning of Bispecific Antibody DNA Constructs

The AUA1 antibody was produced conventionally by immunizing BALB/c mice with the colon adenocarcinoma cell line Lovo. The mouse AUA1 EpCAM antibody was characterized by Dr. Walter Bodmer’s Lab and described in [26]. Humanization of the mouse AUA1 antibody [27] was performed as described in [28,29]. The humanized antibody was first checked for functional activity in the chimeric antigen receptor (CAR) format as described in [29,30]. The humanized variable fragment heavy chain (VH) and light fragment chain (VL) of the AUA1 antibody were used for the engineering of CrossMab knob-in-hole design bivalent antibody constructs as described in [21]. Human Fc (IgG1) contained P329G and L234AL235A (LALA) mutations to silence the Fc region by preventing the binding of gamma receptors and activating innate immune cells [21]. Another design contained an EpCAM ScFv-CD3 ScFv-His tag (BITE) format. The third design was an EpCAM ScFv-CD3 ScFv-human Fc construct. All constructs were cloned into the pYD11 vector and confirmed by sequencing. For the design of DNA templates for RNA-based expression, the EpCAM ScFv-CD3 ScFv-human Fc sequence was cloned into a DNA vector with a T7AG promoter, 5′UTR, 3′UTR, and a 150 poly A tail as described [31]. The DNA template sequence for in vitro transcription was verified by sequencing.

### 2.4. In Vitro Transcription

The mRNA was in vitro transcribed from a DNA template using the HiScribe T7 mRNA Kit with CleanCap Reagent AG (NEB #E2080). In brief, a DNA template, 0.5 × T7 CleanCap Reagent AG Reaction Buffer, 5 mM of ATP, CTP, pseudo-UTP, and GTP were added to 4 mM of CleanCapAG and T7 polymerase mix for 2 h at 37 °C. Then, DNAse I treatment was performed for 15 min at 37 °C. The mRNA was purified with the Monarch RNA Cleanup Kit (T2050) according to the manufacturer’s protocol.

### 2.5. Embedding of mRNA into LNP and Transfection of mRNA-LNP into Cells 

To generate an mRNA–LNP complex, an aqueous solution of mRNA in 100 mM sodium acetate (pH 4.0) was combined with a lipid mix containing the ethanol phase of SM-102 (Cayman), DSPC (Avanti), cholesterol (Sigma), and DMG-PEG2000 (Cayman) (at a molar % ratio of 50:10:38.5:1.5, respectively) at a flow rate ratio of 3:1 (aqueous:organic) using the PreciGenome Flex S System (San Jose, CA, USA). The mRNA-LNPs were purified and concentrated using Amicon^®^ Ultra-15 centrifugal filter units (30–100 kDa). The size, zeta-potential, and polydispersity index (PDI) of the mRNA-LNPs were detected using an Anton Paar Litesizer 500 System, and the encapsulation efficiency was checked with the Quant-it^TM^ RiboGreen RNA assay Kit. The mean mRNA-LNP size was 104 nm; the zeta-potential was −4.8 mV; the PDI was 0.14; and the encapsulation efficiency was 91.8 ± 4.5%. 

In brief, 1 μg of mRNA-LNPs was used to transfect 0.5–1 × 10^6^ HEK-293 cells or other cancer cell lines. The supernatant with secreted EpCAM-CD3 hFc bispecific antibody was collected 48–72 h after transfection, and the correct size was confirmed through Western blotting. The binding with target cells was performed using FACS. 

### 2.6. Transfection of HEK-293 Cells with DNA Encoding Bispecific Antibodies

HEK293S cells were transfected using DNA constructs of the bispecific antibodies with the ALSTEM NanoFect Transfection Reagent. The cells were cultured in Freestyle F17 medium with 8 mM glutamine and 0.1% Pluronic F68 surfactant in suspension bottles using a shaker at 37 °C and 5% CO_2_. Supernatants containing antibodies were collected on days 3–7 posttransfection. The supernatants containing antibodies with human Fc were purified using protein A or protein G columns. The supernatant containing EpCAM-CD3-His tag antibody was purified using Ni-NTA columns. The purified antibodies were checked for size on SDS gel and then used for functional analyses.

### 2.7. Flow Cytometry (FACS)

To measure the binding of the bispecific antibodies, 0.25 × 10^6^ cells were suspended in 0.1 mL of 1 × PBS buffer containing 2 mM EDTA and 0.5% BSA and incubated on ice with 1 µL of human serum (Jackson ImmunoResearch, West Grove, PA, USA) for 10 min. The bispecific antibodies were added to the cells, which were then incubated on ice for 30 min. The cells were washed 3 times with FACS buffer and resuspended in 0.1 mL of buffer. Then, 1 µL of phycoerythrin (PE)-conjugated or APC-conjugated secondary antibody (BD Biosciences, San Jose, CA, USA) was added, and the cells were incubated on ice for 30 min. The antibodies containing human Fc or His Tag were stained with an antihuman-IgG antibody or anti-His tag antibody, respectively, and incubated on ice for 30 min, washed with FACS buffer three times, and stained with PE-streptavidin antibody on ice for 30 min. The cells were washed three times and resuspended in buffer for analysis on an FACSCalibur (BD Biosciences). 

Supernatants containing EpCAM-CD3-hFc antibody (resulting from mRNA-LNP transfection to cancer cells) were collected 48–72 h posttransfection and incubated with the target cells on ice for 30 min. Cells were washed three times with FACS buffer, stained with antihuman-IgG (Fc fragment specific) (Jackson ImmunoResearch), and incubated on ice for 30 min. Cells were washed three times with FACS buffer and resuspended in the buffer for analysis on an FACSCalibur (BD Biosciences).

### 2.8. Real-Time Cytotoxicity Assay (RTCA)

Adherent Lovo (EpCAM^+^) and Colo741 (EpCAM^−^) target cells were seeded in triplicate into 96-well E-plates (Acea Biosciences/Agilent, San Diego, CA, USA) at 1–4 × 10^4^ cells per well overnight using the impedance-based real-time cell analysis (RTCA) xCELLigence system (Acea Biosciences/Agilent). The next day, the medium was removed and replaced with AIM-V medium containing 10% FBS. Effector T cells were added to the target cells at a 10:1 ratio either alone or with different dilutions of antibodies, in triplicate. The cells in the E-plates were monitored for 24 h with the RTCA system, and the impedance was plotted over time. The supernatant was collected after RTCA and used in an ELISA to detect IFN-gamma secretion.

### 2.9. ELISA (Enzyme-Linked Immunoassay)

Target cells were cultured with different dilutions of bispecific antibodies in U-bottom 96-well plates in 200 µL of AIM-V medium containing 10% FBS, in triplicate, and then analyzed with the ELISA to determine human IFN-gamma secretion levels using the R&D Systems Human IFN-gamma Quantikine Kit (Minneapolis, MN, USA) according to the manufacturer’s protocol. 

### 2.10. Mouse Xenograft Tumor Model

Six-week-old NSG mice (Jackson Laboratories, Bar Harbor, ME, USA) were housed and handled in strict accordance with the Institutional Animal Care and Use Committee (#LUM-001) (IACUC) guidelines. Each mouse was injected subcutaneously on day 0 with 100 µL of 2 × 10^6^ OVCAR-5 cells. EpCAM-CD3 hFc mRNA-LNPs were injected intratumorally (1 µg/mice) on certain days, and 1 × 10^7^ T cells were injected either 1 or 3 times intravenously 24–48 h after the injection of mRNA-LNPs. The tumors were measured twice a week with calipers, and the tumor volume was calculated using the following formula: ½ (Length × Width^2^). Tumor growth curves were generated for each group.

### 2.11. Statistical Analysis

Comparison between two groups was performed using the unpaired Student’s *t*-test. Differences with *p* < 0.05 were considered significant. 

## 3. Results

### 3.1. The AUA1 Mouse Antibody Was Humanized and Used to Engineer Three Designs of Humanized AUA1-CD3 Bispecific Antibodies

**Figure 1:**
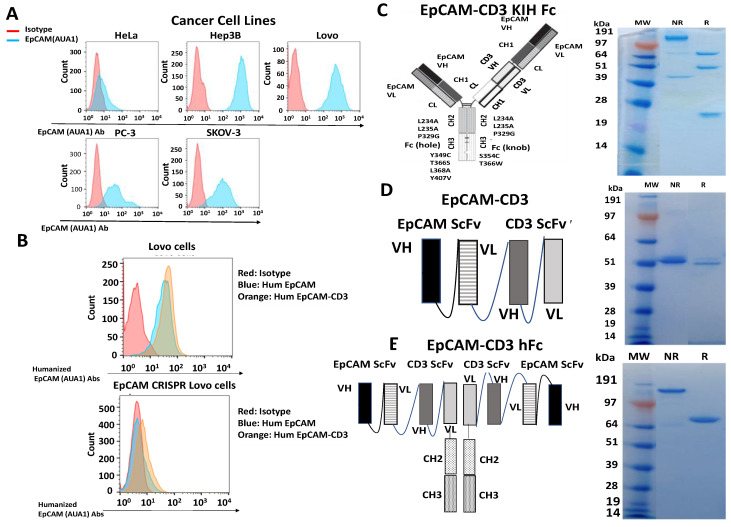
Different designs of humanized EpCAM(AUA-1)-CD3 bispecific antibodies. (**A**) The mouse AUA1 antibody detects EpCAM on different cancer cell surfaces by FACS analysis. This antibody was used for FACS staining with HeLa, Hep3B, Lovo, PC3, and SKOV-3 cells. (**B**) Humanized regular and bispecific humanized EpCAM ScFv-CD3 Scfv antibodies specifically bound to the EpCAM antigen in Lovo cells and did not bind in Lovo cells with CRISPR/Cas-9 knockout of EpCAM (EpCAM KO). (**C**) EpCAM-CD3 CrossMab KIH design and SDS gel for reduced (R) and nonreduced (NR) conditions are shown. (**D**) EpCAM-CD3 (BITE) format design and SDS gel for R and NR conditions are shown. (**E**) EpCAM-CD3 hFc format design and SDS gel for R and NR conditions are shown.

We used the mouse AUA1 antibody [26] for FACS with several different cancer cell lines and detected high binding to different types of cancer cell lines such as cervical cancer HeLa, hepatocellular carcinoma Hep3B, colorectal Lovo, ovarian SKOV-3, and prostate PC3 cancer cell lines (Figure 1A). We humanized the AUA1 antibody and tested binding by FACS using the EpCAM-positive Lovo cancer cell line and the EpCAM knockout (KO) Lovo cell line generated with CRISPR/Cas-9. The humanized EpCAM antibody showed binding to Lovo cells and did not show binding to the Lovo EpCAM-KO cell line (Figure 1B). This demonstrates the high specificity of the humanized AUA1 antibody. 

We used this humanized antibody to generate bispecific antibodies using three different designs: bivalent EpCAM-CD3 CrossMab knob-in-hole with silenced Fc, EpCAM-CD3 KIH Fc (Figure 1C); EpCAM ScFv-CD3 ScFv-His tag (BITE) (Figure 1D); and EpCAM ScFv-CD3 ScFv human Fc (Figure 1E). All antibodies were purified, checked for correct size on SDS gel, and used for functional analysis with FACS, real-time cytotoxicity assay (RTCA), and IFN-gamma secretion.

### 3.2. EpCAM-CD3 Bispecific Antibodies Caused High Binding, Killing, and IFN-Gamma Secretion with EpCAM-Positive Target Cancer Cells

**Figure 2:**
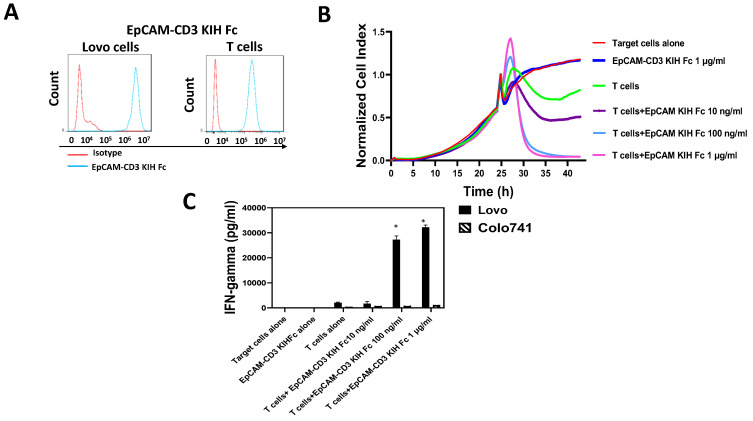
EpCAM-CD3 CrossMab KIH antibody specifically binds to EpCAM-positive cells and T cells, kills EpCAM-positive target cells, and induces IFN-gamma secretion with T cells. (**A**) FACS shows binding of bispecific antibody to EpCAM-positive Lovo cells and to T cells. (**B**) Real-time cytotoxicity (RTCA) assay demonstrates killing of EpCAM-positive Lovo cells by EpCAM CrossMab KIH-CD3 antibody with T cells, in a dose-dependent manner. (**C**) EpCAM CrossMab KIH-CD3 antibody induces IFN-gamma secretion by T cells against EpCAM-positive cells. There was no induction of IFN-gamma secretion against EpCAM-negative Colo741 cells. * *p* < 0.05, Student’s *t*-test IFN-gamma secretion against Lovo cells versus Colo741 cells.

We tested bivalent EpCAM-CD3 CrossMab KIH Fc for binding to EpCAM-positive Lovo cells and CD3-positive T cells (Figure 2A) using FACS. The antibody showed binding to Lovo cells and T cells (Figure 2A) and did not show binding to EpCAM-negative Colo741 cells (not shown). The EpCAM-CD3 CrossMab KIH Fc killed Lovo cells when combined with T cells in a dose-dependent manner using the Agilent RTCA system (Figure 2B). The supernatant was collected and tested for the secretion of IFN-gamma by T cells, which showed high levels of secretion with Lovo target cells and did not show any increase in IFN-gamma secretion with Colo741 target cells (Figure 2C). Thus, EpCAM-CD3 CrossMab KIH Fc has high dose-dependent efficacy against EpCAM-positive Lovo cells.

**Figure 3:**
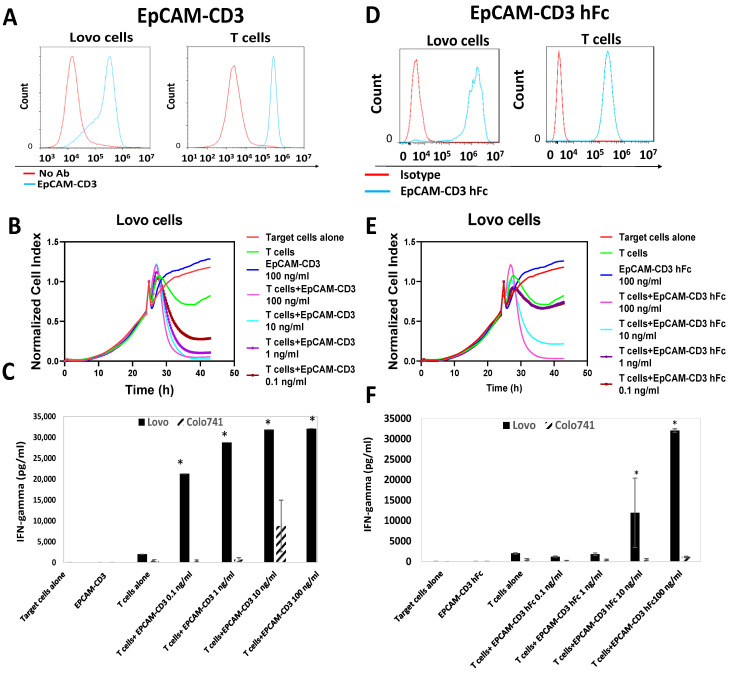
EpCAM-CD3 and EpCAM-CD3 human Fc antibodies bind to EpCAM-positive cells and T cells, kill EpCAM-positive target cells, and induce IFN-gamma secretion with T cells. (**A**) FACS shows binding of EpCAM-CD3 antibody to EpCAM-positive Lovo cells, as well as T cells. (**B**) RTCA assay demonstrates dose-dependent killing of Lovo target cells by antibody when incubated with T cells. (**C**) EpCAM-CD3 antibody with T cells induces IFN-gamma secretion against Lovo cells but not against Colo741 cells. (**D**) FACS shows binding of EpCAM-CD3 human Fc antibody to Lovo and T cells. (**E**) RTCA assay demonstrates the dose-dependent killing of EpCAM-positive target cells by antibody when incubated with T cells. (**F**) EpCAM-CD3 hFc antibody with T cells induces IFN-gamma secretion against Lovo cells and not against Colo741 cells. * *p* < 0.05, Student’s *t*-test. IFN-gamma secretion increased when the antibody was incubated with T cells against Lovo cells and not against Colo741 cells.

Next, we tested the EpCAM-CD3 (BITE) antibody for binding to Lovo and T cells using FACS (Figure 3A). The antibody showed high binding to Lovo and T cells (Figure 3A). The antibody combined with T cells killed Lovo target cells (Figure 3B) and did not kill Colo741 cells (not shown). The EpCAM-CD3 antibody combined with T cells showed a high level of secreted IFN-gamma with Lovo target cells and did not show it with EpCAM-negative Colo741 target cells (Figure 3C).

Next, we tested the EpCAM-CD3 hFc antibody for binding to Lovo and T cells using FACS, killing activity, and IFN-gamma secretion. This antibody showed high binding to EpCAM-positive Lovo cells and T cells (Figure 3D), effectively killed Lovo cells (Figure 3E), and did not kill Colo741 cells (not shown). The EpCAM-CD3 hFc incubated with T cells caused the secretion of IFN-gamma against Lovo cells and not against Colo741 cells (Figure 3F). The antibody alone without T cells did not cause the secretion of IFN-gamma (Figure 3F). Thus, all three bispecific antibody designs had high and specific in vitro activity against EpCAM-positive cells.

### 3.3. EpCAM-CD3 hFc mRNA-LNPs Transfected in HEK293 Cells Produce EpCAM-CD3 hFc Antibody with Highly Specific Functional Activity against EpCAM-Positive Cells

**Figure 4:**
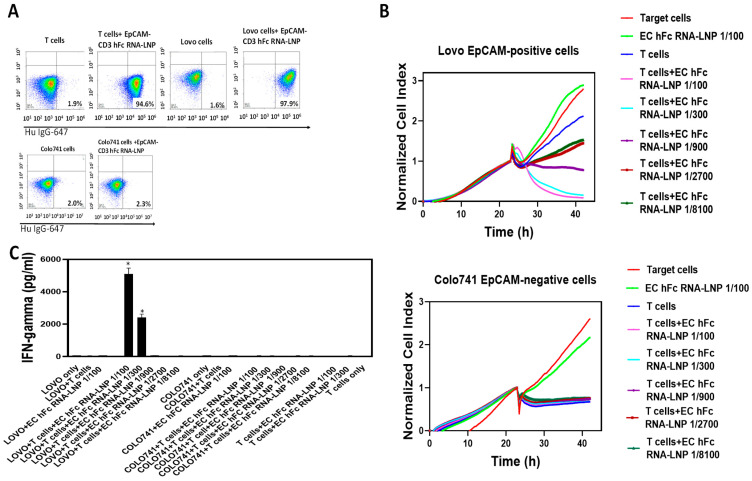
EpCAM-CD3 hFc mRNA-LNP transfection to HEK-293 cells generates antibodies that bind to Lovo and T cells and demonstrates specific killing. (**A**) FACS of Lovo, Colo741, and T cells is shown with supernatant collected from HEK-293 cells transfected with mRNA-LNPs. (**B**) RTCA assay of Lovo and Colo741 cells using supernatant at different dilutions collected from mRNA-LNP-transfected HEK-293 cells. RTCA demonstrates dose-dependent killing of target cells by antibody and T cells. As a negative control, supernatant from untransfected HEK-293 cells was used and tested in an RTCA assay at the same dilutions as the supernatant from transfected HEK-293 cells. (**C**) IFN-gamma secretion by T cells with EpCAM-CD3 hFc supernatant collected after RTCA against Lovo and Colo741 target cells. The secretion of IFN-gamma was significantly higher for Lovo target cells than for Colo741 target cells. * *p* < 0.05, Student’s *t*-test, EpCAM-CD3 hFc with T cells against Lovo cells versus the same conditions against Colo741 cells.

To test the production of antibodies using mRNA, we used an EpCAM-CD3 hFc DNA design and subcloned this antibody-encoding sequence into a DNA template for in vitro transcription with a T7AG promoter, 5′UTR, 3′UTR, and a 150 poly A tail. This template was used for in vitro transcription to generate mRNA for this antibody. The mRNA was embedded into LNPs using the PreciGenome Flex S microfluidic system. EpCAM-CD3 hFc mRNA-LNPs were transfected into HEK-293 cells, and 48–72 h later, the supernatant containing antibodies was collected and used for functional assays. The supernatant showed binding to Lovo and T cells but did not bind to EpCAM-negative Colo741 cells (Figure 4A). Dose-dependent killing of Lovo cells but not Colo741 cells was observed with the collected supernatant (Figure 4B). This was also accompanied by T cell secretion of IFN-gamma with Lovo target cells and not with Colo741 target cells (Figure 4C).

Thus, transfection of the EpCAM-CD3 hFc mRNA-LNPs to HEK293 cells produced functional antibodies that showed binding to EpCAM-positive target cells and demonstrated highly specific killing activity and secretion of IFN-gamma when combined with T cells against EpCAM-positive target cells. 

### 3.4. EpCAM-CD3 hFc mRNA-LNPs Transfected to Cancer Cells Produced Bispecific Antibody with Specific Functional Activity against EpCAM-Positive Cells

**Figure 5:**
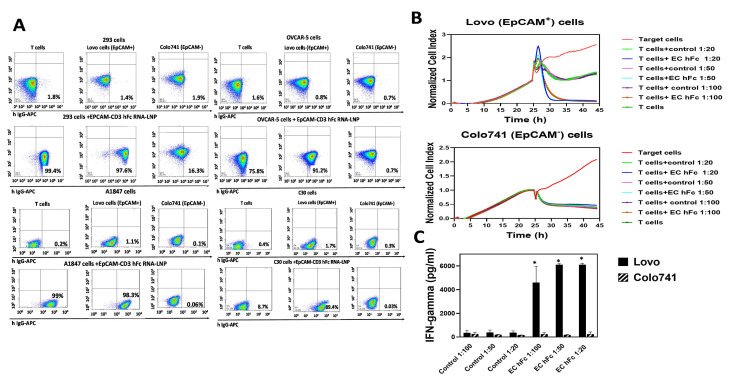
OVCAR-5, A1847, and C30 cancer cell lines secrete functional EpCAM-CD3-hFc antibody after mRNA-LNP transfection. (**A**) FACS with supernatant collected from EpCAM-CD3 hFc mRNA-LNP transfected OVCAR-5, A1847, and C30 cells shows high binding of secreted antibodies to EpCAM-positive Lovo and T cells and shows no binding to Colo741 cells. (**B**) RTCA assay shows that supernatant collected 48 h after EpCAM-CD3 hFc mRNA-LNP transfection of OVCAR-5 cells kills target Lovo cells in the presence of T cells and does not kill Colo741 cells under the same conditions. No killing was observed for the supernatant containing the EpCAM-CD3 hFc antibody alone against target cells without T cells. (**C**) Induction of IFN-gamma secretion by T cells detected in the supernatant collected after RTCA assay (**B**) for Lovo target cells and not for Colo741 target cells. * *p* < 0.05, Student’s *t*-test, IFN-gamma secretion against Lovo cells versus Colo741 cells.

Next, we wanted to check whether cancer cells transfected with EpCAM-CD3 hFc mRNA-LNPs would produce functional antibodies. We transfected ovarian and colorectal cancer cell lines and detected that OVCAR-5 colorectal and ovarian A1847 and C30 cancer cell lines produced antibodies that showed binding to Lovo cells and T cells and did not show binding to Colo741 cells (Figure 5A). The antibodies generated from the OVCAR-5 cells killed Lovo cells with added T cells in a dose-dependent manner (Figure 5B) and induced the secretion of IFN-gamma by T cells (Figure 5C).

Thus, cancer cell lines can be transfected with EpCAM-CD3 hFc mRNA-LNPs and produce functional antibodies with specific EpCAM-dependent activity against cancer cells.

### 3.5. EpCAM-CD3 hFc mRNA-LNPs with T Cells Significantly Blocked OVCAR-5 Xenograft Tumor Growth

**Figure 6:**
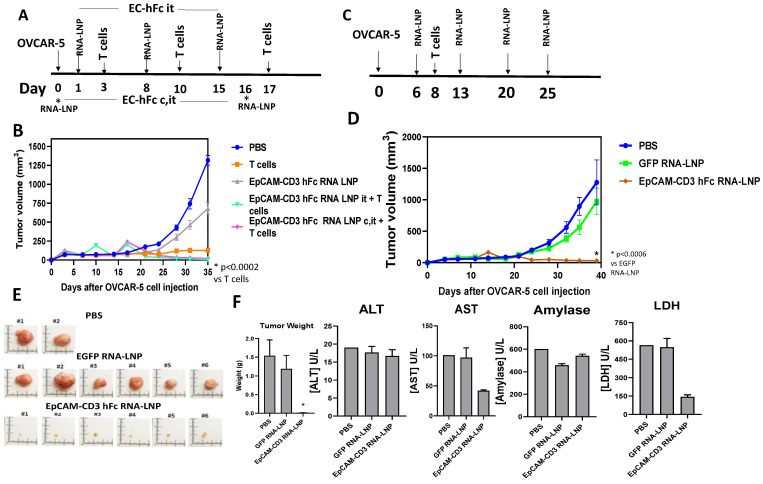
EpCAM-CD3 hFc mRNA-LNPs delivered to OVCAR-5 tumors with intravenous injection of T cells significantly decreased OVCAR-5 xenograft tumor growth. (**A**) The schedule for mRNA-LNP intratumoral (i.t.) injections and three T cell intravenous (i.v.) injections to OVCAR-5 tumor groups. Note: c. marks cellular delivery of EpCAM-CD3-hFc mRNA-LNP pre-mixed with OVCAR-5 cancer cells; i.t. marks intratumoral delivery; i.v. marks intravenous delivery of T cells. (**B**) EpCAM-CD3 hFc mRNA-LNPs combined with T cells significantly decreased OVCAR-5 xenograft tumor growth. (**C**) The schedule for mRNA-LNP intratumoral injection and a single injection of T cells i.v. delivered to OVCAR-5 xenograft tumor model. (**D**) EpCAM-CD3 hFc mRNA-LNPs significantly decreased OVCAR-5 tumor growth combined with a single injection of T cells. (**E**) Tumor size and weight significantly decreased with EpCAM-CD3-hFc mRNA-LNP and T cell treatment. Images of tumors (left panel) are shown at the end of experiment. Bars with averages of tumor weight are shown in the right panel. For tumor weights: * *p* < 0.05, EpCAM-CD3 hFc mRNA-LNP group versus PBS and GFP mRNA-LNP groups, Student’s *t*-test. (**F**) There was no increase in ALT, AST, amylase, or LDH levels in mouse serum collected at the end of treatment for the EpCAM-CD3-hFc mRNA-LNP group versus the GFP mRNA-LNP group for the OVCAR-5 xenograft tumor model.

OVCAR-5 cells were injected subcutaneously into NSG mice, and then, one group of mice were injected at days 1, 8, and 15 intratumorally (i.t. group) with EpCAM-CD3 hFc mRNA-LNPs and then with T cells injected intravenously on days 3, 10, and 17 (Figure 6A). Another group (c, i.t. group) of mice were injected with OVCAR-5 cells pre-mixed together with EpCAM-CD3 hFc mRNA-LNPs (cellular injection, c.), and then, for a second time they were injected with EpCAM-CD3 hFc mRNA-LNPs on day 16 intratumorally (i.t.), and T cells were injected intravenously on the same days as above (Figure 6A). Both treatments of EpCAM-CD3 hFc mRNA-LNPs delivered to mice combined with T cells significantly decreased OVCAR-5 tumor growth (Figure 6B). The inhibition of tumor growth was significantly higher (*p* < 0.05) with EpCAM-CD3 mRNA-LNPs and T cell delivery than with T cells alone. EpCAM-CD3 hFc mRNA-LNPs alone without T cells did not block tumor growth (Figure 6B). Thus, the high antitumor activity of EpCAM-CD3 hFc mRNA-LNPs combined with T cells was demonstrated in vivo.

Next, we tested EpCAM-CD3 hFc mRNA-LNPs delivered intratumorally on days 6, 13, 20, and 25 with only a single injection of T cells i.v. on day 8 (Figure 6C). As a negative control, we used GFP mRNA-LNPs. The EpCAM-CD3 mRNA-LNPs significantly blocked OVCAR-5 xenograft tumor growth while the GFP mRNA-LNPs did not (Figure 6D). The images of tumors at the end of treatment are shown in Figure 6E. There was significant reduction in tumor size and weight in the EpCAM-CD3 hFc mRNA-LNP and T cell–treated group versus the PBS and GFP mRNA-LNP–treated group. In addition, we collected serum from the treated mice and showed that there was no increase in blood toxicology markers (AST, ALT, amylase, and LDH) caused by EpCAM-CD3 hFc mRNA-LNP treatment versus GFP mRNA-LNP treatment suggesting no toxicity of the EpCAM-CD3 mRNA-LNPs (Figure 6F). AST and LDH levels were significantly decreased by EpCAM-CD3 hFc mRNA-LNP versus GFP mRNA-LNP treatment (Figure 6F).

Thus, EpCAM-CD3 hFc mRNA-LNPs can produce antibodies inside cancer cells with high antitumor efficacy in vivo.

## 4. Discussion

We tested three different designs of humanized EpCAM-CD3 antibodies such as bivalent CrossMAB knob-in-hole KIH Fc, EpCAM-CD3 (BITE), and EpCAM-CD3-hFc. All the antibodies demonstrated highly specific binding and killing activity against EpCAM-positive target cells and not against EpCAM-negative cells. In addition, we developed mRNA-LNP technology to produce EpCAM-CD3 hFc antibodies that showed highly specific anticancer efficacy in vitro in HEK-293 cells and in various cancer cells. The delivery of mRNA-LNPs into mice tumors with intravenous T cell significantly blocked OVCAR-5 xenograft tumor growth in vivo.

This study demonstrates a novel method of delivering bispecific antibodies using mRNA-LNP technology. The mRNA-LNP technology platform was recently used for COVID-19 vaccines by Moderna and Pfizer and was proven to be safe, which provides a solid basis for future anticancer vaccine, antibody production, and novel therapeutics development. 

The production of efficacious antibodies was shown by the intratumoral delivery of EpCAM-CD3 hFc mRNA-LNPs. This delivery was shown to be safe as there was no increase in toxicology blood markers such as ALT, AST, amylase, and LDH in the collected serum of treated mice. The encoding of antibodies and drugs through mRNA has been discussed using nanotechnology and oncolytic viruses [20,32]. This study shows nonviral delivery of mRNA-LNP to tumors.

We also tested the same EpCAM-CD3 antibody with mutant, silenced Fc as was shown for the CrossMab KIH design to decrease potential immune activation through NK receptors. The EpCAM-CD3 mutant Fc antibody generated with mRNA-LNP technology demonstrated the same high efficacy as the wild-type Fc antibody (not shown). 

The production of bispecific antibodies through mRNA-LNP injection in vivo has many advantages versus RNA or antibody proteins. The mRNA–LNP complexes are more stable than regular mRNA, and the production of antibodies through mRNA-LNPs is less costly than antibody protein manufacturing and shows potential for further optimizations in future studies. 

This novel approach included bispecific antibody generation using safe and local intratumoral delivery using mRNA-LNP technology. Moreover, it combined a cell therapy approach by using T cells to kill tumors. This approach can be used similarly to attract NK cells or gamma–delta T cells to tumors using specific immune cell receptors. In addition, different stimulators of immune cells can be used to increase the efficacy of this therapy such as checkpoint inhibitor players, chemokines, cytokines, growth factors, and tumor microenvironment modulators. The lysed tumor releases neoantigens (natural vaccine), which can be recognized in the presence of immunomodulators by antigen-presenting cells or dendritic cells and promote the activity of memory T and B cells. Thus, this approach can be developed and optimized for future clinical applications.

## 5. Conclusions

In conclusion, EpCAM-CD3 human Fc bispecific antibodies generated with mRNA-LNP technology demonstrate high efficacy in vitro and in vivo. This study, for the first time, shows that intratumoral delivery of EpCAM-CD3 hFc mRNA-LNPs with intravenous delivery of T cells blocked xenograft tumor growth. This approach can be used against solid tumors in future clinical studies.

## 6. Patents

The antibody sequences are included in the patent application.

## Data Availability

Data are contained within the article.
